# Dictionary-Augmented Large Language Model Postprocessing for Bilingual Code-Switched Medical Speech Recognition: Development and Evaluation Study

**DOI:** 10.2196/91696

**Published:** 2026-07-08

**Authors:** Chanryeong Oh, Yul Hwangbo, Wonjoong Cheon, Jaedong Lee

**Affiliations:** 1 Healthcare AI Team, National Cancer Center, Goyang-si, Gyeonggi-do, 10408, Republic of Korea, 821075293175; 2 Department of Public Health & AI, Graduate School of Cancer Science and Policy, National Cancer Center, Goyang-si, Gyeonggi-do, Republic of Korea; 3 Department of Radiation Oncology, Seoul St. Mary's Hospital, College of Medicine, The Catholic University of Korea, 222, Banpo-daero, Seocho-gu, Seoul, 06591, Republic of Korea, 821045145249

**Keywords:** automatic speech recognition, code-switching, medical terminology, large language model, natural language processing

## Abstract

**Background:**

Clinical documentation burden contributes significantly to physician burnout, with health care professionals spending much of their time on electronic health record interactions. Automatic speech recognition (ASR) systems offer a promising solution; however, their application in Korean medical settings faces unique challenges due to widespread Korean-English code-switching, where clinicians routinely alternate between Korean conversational language and English medical terminology within single utterances.

**Objective:**

This study aimed to develop and evaluate a hybrid postprocessing approach combining medical terminology dictionary normalization with large language model (LLM)–based postprocessing to improve ASR accuracy for Korean-English code-switched medical speech.

**Methods:**

We constructed a speech dataset from 23,652 nursing progress notes, with a linguistic composition of 67.73% (512,626/756,866) Korean, 23.54% (178,166/756,866) English, and 8.73% (66,074/756,866) numerals or special symbols. Four Korean nurses recorded the notes using 5 microphone types in an acoustically isolated environment. Speech recognition was performed using OpenAI’s gpt-4o-transcribe model. For postprocessing, a medical terminology dictionary containing 1070 mapping entries was constructed from 1000 nursing progress notes to normalize Korean phonetic renderings of English medical terms. Six LLMs (2 GPT and 4 Claude variants) were then evaluated across 5 temperature settings (0.0‐0.8). Performance was assessed using BERTScore (bidirectional encoder representations from transformers score; *F*_1_), Sentence-BERT cosine similarity, word error rate, and character error rate (CER), comparing postprocessed outputs against the original written notes. Statistical significance was assessed using paired Wilcoxon signed-rank tests with Holm correction (α=.05).

**Results:**

Temperature optimization showed that all postprocessing models had small temperature-related effect sizes (all |Cohen *d*_z_| ≤0.15), with GPT-4o exhibiting the largest dependency and statistically significant improvement at temperature 0.6 (Holm-adjusted *P*<.001 for both BERTScore and CER) and the 4 Claude variants and GPT-4.1 exhibiting practically consistent performance across all settings. Baseline ASR achieved a BERTScore of 0.9131 and CER of 0.2336. Dictionary-based normalization performed 43,507 word-level substitutions in 70.8% (16,754/23,652) of transcribed sentences. LLM-only postprocessing reduced CER by 36.09% (Claude Sonnet 4) and 32.53% (GPT-4o) compared to baseline. The combined dictionary-LLM approach achieved the best performance: Claude Sonnet 4 attained a BERTScore of 0.9638 and CER of 0.0820, representing a 64.9% reduction in CER from baseline (*P*<.001).

**Conclusions:**

The hybrid pipeline integrating rule-based dictionary normalization with LLM postprocessing significantly improved Korean-English code-switched medical ASR accuracy. Dictionary-based normalization yielded consistent additional improvements over LLM-only postprocessing for both GPT-4o and Claude Sonnet 4. As this modular framework requires no model retraining, it offers a practical means of mitigating multilingual challenges in medical ASR.

## Introduction

Health care systems worldwide face an escalating physician burnout crisis that directly threatens patient care quality and system sustainability. Recent studies indicate that more than 60% of physicians report experiencing burnout symptoms, with administrative documentation burden identified as a primary contributing factor [1,2]. Health care systems worldwide are increasingly vulnerable to workforce crises—the Republic of Korea recently experienced nationwide physician strikes that disrupted patient care and research output [3]—underscoring the urgency of addressing modifiable factors such as documentation burden.

The widespread adoption of electronic health records (EHRs) has fundamentally transformed clinical documentation practices. While EHRs offer benefits such as enhanced data accessibility and secure long-term storage, documentation requirements have escalated, with primary care physicians now spending nearly half of their workday interacting with EHR systems [4]. To alleviate this burden, automatic speech recognition (ASR) systems have emerged as one of the most promising solutions among various artificial intelligence technologies [5,6].

Recent advances in deep learning architectures, particularly Transformer-based models, have driven significant improvements in ASR performance across diverse linguistic contexts [7,8]. Large-scale pretrained ASR systems have emerged as a dominant paradigm. OpenAI’s Whisper Large-v3, trained on more than 5 million hours of multilingual audio data, and Google’s Chirp models, trained on millions of hours spanning more than 100 languages, exemplify this trend [9,10]. These foundation models represent a paradigm shift from language-specific supervised training to large-scale self-supervised learning.

The convergence of these technological capabilities with the urgent need for clinical documentation solutions has created new possibilities for ASR deployment in health care settings. However, existing medical ASR approaches face inherent limitations: domain-specific models, while outperforming general-purpose systems in overall accuracy, continue to struggle with precise terminology recognition and exhibit poor generalizability across clinical specialties and institutions [11,12]. The acoustic variability in clinical environments and the critical need for accuracy in safety-sensitive applications further compound these challenges.

Comparative studies of commercial ASR engines have revealed considerable variability in medical concept recognition. Specialized terms and abbreviations are frequently misrecognized even in domain-adapted systems [12-14]. Additionally, LLM-based postprocessing approaches are susceptible to hallucination phenomena despite their contextual understanding capabilities. This poses serious risks in clinical environments where factual accuracy is critical for patient safety [15].

The challenge is intensified in multilingual clinical environments where code-switching between languages, such as Korean and English, is routine. In these settings, weaknesses in existing ASR systems are exposed: inconsistent recognition at language transition points and inadequate handling of domain-specific bilingual expressions [16-18]. These challenges necessitate new approaches that integrate the strengths of different methodologies while mitigating their weaknesses.

The Korean health care environment presents particularly complex challenges for ASR implementation due to the widespread use of Korean-English code-switching in clinical discourse. Korean medical professionals routinely alternate between Korean conversational language and English medical terminology within single utterances, creating frequent language transitions that confound conventional multilingual ASR systems [19]. Previous research has addressed Korean-English code-switching in ASR, including end-to-end approaches using medical record datasets [20] and phonetic variation modeling for Korean-style English pronunciation [21]. However, these studies primarily focused on general code-switching scenarios rather than the specific challenges of clinical documentation, which demands precise recognition of specialized medical terminology. This code-switching pattern differs significantly from general bilingual speech in that it involves highly specialized medical vocabulary, standardized abbreviations, and domain-specific expressions that require precise recognition for clinical accuracy. Moreover, the Korean medical education system’s emphasis on English medical terminology has resulted in pronunciation patterns and accent variations that are not adequately represented in existing ASR training datasets. Current multilingual ASR models, while capable of handling isolated Korean or English speech, demonstrate degraded performance at code-switching boundaries where critical medical information is often communicated. These linguistic complexities, compounded by the acoustic challenges of clinical environments, necessitate specialized approaches that can accurately handle this unique pattern of medical code-switching while maintaining the high accuracy standards essential for clinical documentation.

While recent studies have explored LLM-based postprocessing for medical ASR [22,23], the specific application to Korean-English code-switching environments remains underexplored. To address this gap, this study proposes a hybrid postprocessing approach integrating dictionary-based terminology normalization with LLM-based postprocessing. This methodology combines the precision of curated dictionaries with the contextual capabilities of LLMs to improve transcription accuracy in code-switched clinical discourse. The effectiveness of this integrated approach is evaluated through comparative analysis of multiple processing strategies and semantic similarity metrics.

## Methods

### Study Design

We evaluated the effectiveness of postprocessing approaches for Korean-English code-switched medical ASR through three sequential phases: (1) establishing baseline ASR performance using gpt-4o-transcribe (OpenAI) with temperature optimization, (2) assessing 2 postprocessing strategies—dictionary-based normalization and LLM-based postprocessing—both independently and in combination, and (3) comparing 6 LLMs (2 GPT and 4 Claude variants [Anthropic]) as the postprocessing model under temperature optimization. Across all phases, outputs were evaluated against the original written nursing notes using both semantic similarity (BERTScore and Sentence-BERT cosine similarity) and surface-form error metrics (word error rate [WER] and character error rate [CER]). This framework targets the primary challenge of Korean phonetic renderings of English medical terms in ASR output, with the goal of achieving practically useful transcription accuracy for clinical documentation, subject to further validation.

### Dataset

This ASR study used a speech dataset constructed by having 4 Korean nurses (all female, with 3‐10 years of clinical experience) read deidentified nursing progress notes in an acoustically isolated environment. To simulate the diversity of recording devices encountered in real clinical settings, 5 types of microphone setups were used: a lavalier microphone (BY-M1, BOYA), a gooseneck desktop stand microphone (PLM-401U, PLEOMAX), and a handheld dictation microphone (SpeechMike Premium, Philips) as wired devices; and a bone-conduction headset with boom microphone (OpenComm, Shokz) and a Bluetooth earset (Voyager 5200, Poly) as wireless devices. Each nurse recorded using all 5 microphone types in a rotating daily schedule, ensuring balanced representation across all device-speaker combinations. This design aimed to evaluate ASR robustness under heterogeneous acoustic conditions representative of clinical practice. All audio files were stored in WAV format with a sampling rate of 16 kHz and a mono channel configuration.

The source material for these recordings consisted of 23,652 nursing progress notes randomly selected from approximately 88,000 records documented for hospitalized patients with cancer at the National Cancer Center in Korea between 2019 and 2021. Nursing progress notes represent a type of clinical documentation prepared by nurses to record the progress of inpatients, including medication administration and nursing procedures, and are typically written on a minute-by-minute basis. These original written notes serve as reference transcripts for ASR evaluation. As part of a medical data smart curation process, the source nursing notes were systematically deidentified, quality-checked, and linguistically analyzed to ensure terminological consistency, while preserving the original clinical content as ground-truth references. This process was designed to enhance data reliability and suitability for downstream ASR and language model evaluation without introducing task-specific bias.

All 23,652 notes were used for the final performance evaluation. Within this set, a subset of 1000 notes was additionally used as a development set for dictionary construction (the “Medical Terminology Dictionary” section) and temperature selection (the “LLM-Based Postprocessing” section); this subset was not held out from the evaluation.

Due to the inherent characteristics of clinical documentation, these records contain a variety of medical terminologies, abbreviations, and drug names and are written in a mixed format that combines Korean and English expressions. The collected sentences ranged from 3 to 647 tokens in length, with a mean of 32 (SD 19) tokens. Across 756,866 tokens, the linguistic composition consisted of 512,626 (67.73%) Korean, 178,166 (23.54%) English, and 66,074 (8.73%) numerals or special symbols.

### Speech Recognition Model

This study used OpenAI’s gpt-4o-transcribe (OpenAI Python SDK v1.68.2) model for automatic speech recognition. This model is a GPT-4o–based speech recognition system designed to handle multilingual audio input with automatic language detection capabilities.

For the Korean clinical context, the model was configured with a persona setting as a Korean nurse to better align with domain-specific speech patterns and terminology usage (Multimedia Appendix 1). The language parameter was not specified, allowing the model to automatically detect languages in code-switched utterances. However, because the input audio was predominantly Korean, the model tended to transcribe English medical terms as Korean phonetic renderings rather than standard English forms (eg, “portable” → “포터블”), necessitating subsequent postprocessing for terminology normalization. Temperature parameter optimization was conducted on the development subset across 5 discrete levels (0.0, 0.2, 0.4, 0.6, and 0.8) to determine the optimal setting for transcription consistency.

Given the large volume of audio files (N=23,652), a batch processing approach was implemented with 500 files per batch, resulting in 48 sequential processing batches to ensure computational efficiency and system stability. Each batch required approximately 20 minutes to process.

### Postprocessing Pipeline

To address the issue of Korean phonetic renderings in ASR output, a 2-stage postprocessing pipeline was developed. First, a medical terminology dictionary (the “Medical Terminology Dictionary” section) performs systematic normalization of predictable transcription patterns through rule-based matching. Second, LLM-based postprocessing (the “LLM-Based Postprocessing” section) applies contextual refinement to handle complex variations while preserving the original sentence structure. These complementary approaches (the “Combined Approach” section) are designed to improve both the consistency and accuracy of medical terminology recognition.

### Medical Terminology Dictionary

A medical terminology dictionary was constructed from the development subset to enable systematic normalization prior to LLM postprocessing. The original written notes, which contain English medical terminology, served as reference sentences. The corresponding ASR outputs served as candidate sentences; while some English terms were correctly transcribed, others were rendered as Korean phonetic transcriptions depending on the model’s automatic language detection. The construction procedure was as follows:

1. Each reference-candidate sentence pair was aligned and tokenized based on whitespace.
2. English medical terms (eg, dressing and mepilex border) were identified in the original written notes, and their token positions were recorded.
3. For each identified English term, adjacent tokens within a ±1 position range were extracted from the corresponding ASR output to accommodate positional variations caused by irregular Korean spacing.
4. The Korean phonetic rendering corresponding to each English medical term was manually selected from the extracted ASR tokens to form mapping pairs (eg, “포터블” → “portable,” Table 1).
5. The finalized mapping pairs were compiled into a structured dictionary containing 1070 entries. Representative examples of these mappings are presented in Table 1.

**Table 1.** Representative examples of dictionary mapping from Korean phonetic renderings to English medical terms.

Korean phonetic words	English medical terms
업노말	Abnormal
앰뷸레이션	Ambulation
카테터	Catheter
헤마토마	Hematoma
라파로스코피	Laparoscopy
포터블	Portable

Dictionary-based normalization was performed by comparing every token in the ASR output against the dictionary and replacing matched Korean phonetic tokens with their corresponding English medical terms. Considering the flexible spacing rules and orthographic variations of the Korean language, a 5-step hierarchical matching scheme was applied: (1) exact match, (2) substring match, (3) n-gram match, (4) space-agnostic match, and (5) single-character match. If none of these criteria were satisfied, the original token was retained. All replaced terms were logged in a separate table for postanalysis and validation purposes.

### LLM-Based Postprocessing

An LLM-based postprocessing procedure was applied to normalize Korean phonetic renderings of English medical terms into their standard Romanized equivalents. Six models were evaluated: GPT-4o (gpt-4o-2024-08-06) and GPT-4.1 (gpt-4.1-2025-04-14), both accessed via the OpenAI Python SDK (v1.68.2), and 4 Claude variants accessed via Amazon Bedrock (anthropic.claude-3‐5-sonnet-20241022-v2:0, anthropic.claude-3‐7-sonnet-20250219-v1:0, anthropic.claude-sonnet-4‐20250514 v1:0, and anthropic.claude-opus-4‐20250514 v1:0). These models were selected for practical relevance to clinical deployment: GPT-4o and GPT-4.1 were the most widely adopted OpenAI models at the time of the study, and the 4 Claude variants allowed comparison across model generations (3.5 → 3.7 → 4) and across tiers within a generation (Sonnet vs Opus). GPT-4o was additionally chosen for consistency with the ASR model (gpt-4o-transcribe), which shares the same GPT-4o foundation.

Prompts were developed and optimized separately for each model family, while the processing pipeline was held identical across models to ensure a fair comparison. Both GPT models used a common GPT-optimized prompt (Multimedia Appendix 2), whereas the 4 Claude variants used a Claude-optimized prompt (Multimedia Appendix 3). Each prompt conveyed the same three core instructions: (1) substitution of Hangul phonetic expressions with standardized medical terms or abbreviations; (2) prohibition of translation, summarization, or paraphrasing to preserve the original sentence structure; and (3) explicit conversion rules for drug names, dosage units, and medical abbreviations.

Temperature optimization was performed on the same development subset across the 5 levels (0.0, 0.2, 0.4, 0.6, and 0.8), and the best-performing setting per model was applied to the full dataset for final evaluation. For each model, only temperature was tuned, with top_p (and top_k, where applicable) held at provider-default values [24,25]

### Combined Approach

**Figure 1.**
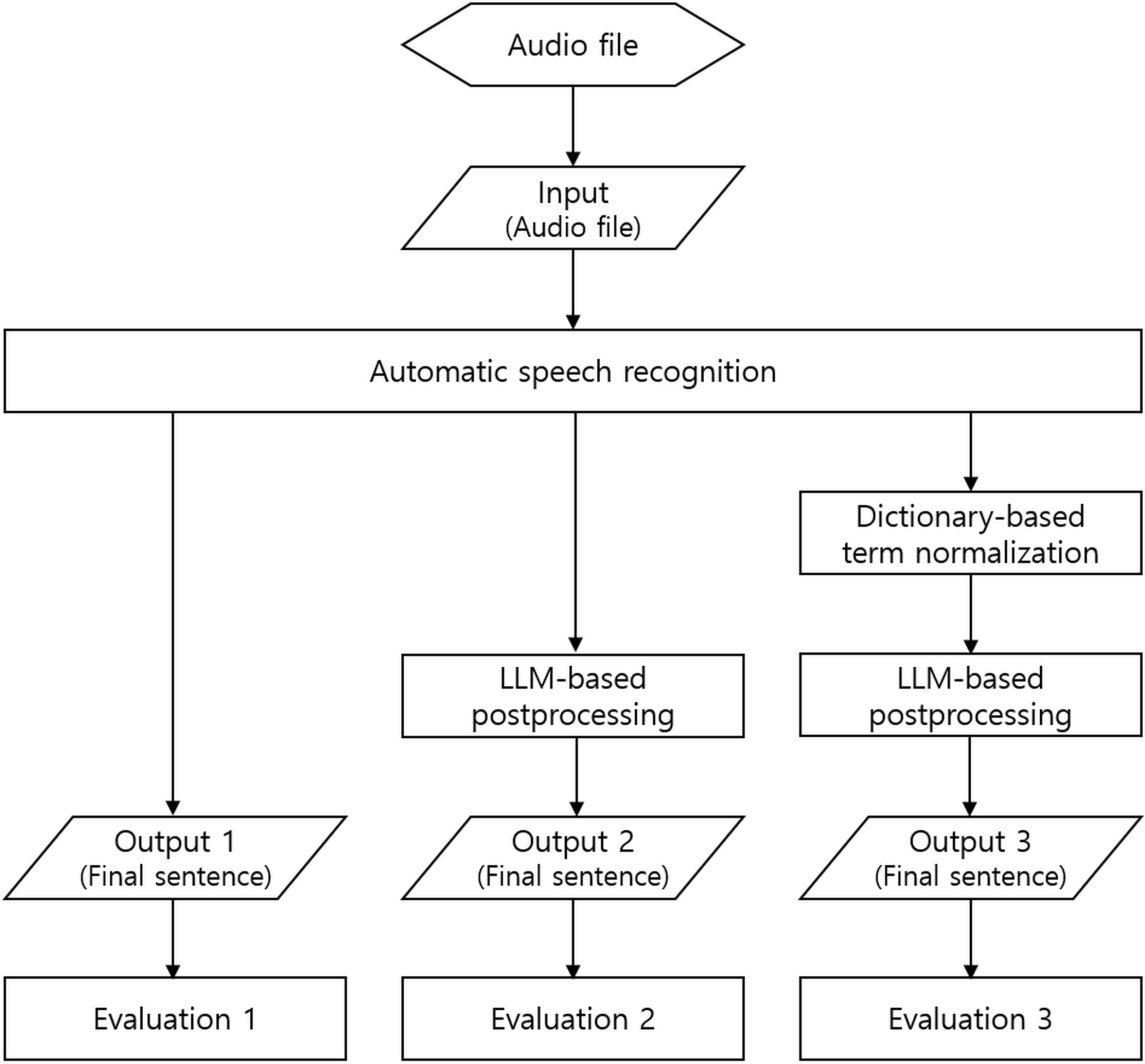
Experimental pipeline comparing baseline automatic speech recognition, large language model–only postprocessing, and dictionary-augmented large language model postprocessing. LLM: large language model.

The complete pipeline was evaluated in three configurations: (1) raw ASR output without postprocessing, (2) ASR with LLM-based postprocessing only, and (3) ASR with dictionary-based normalization followed by LLM postprocessing (Figure 1). This sequential approach combines the systematic correction capabilities of the medical dictionary with the contextual refinement of LLMs, aiming to address both predictable terminology patterns and context-dependent variations in code-switched medical discourse.

### Evaluation Metrics

To quantitatively evaluate the postprocessing pipeline, 4 complementary metrics were adopted: BERTScore (*F*_1_), Sentence-BERT (SBERT) cosine similarity, WER, and CER. All metrics compared the postprocessed ASR outputs (hypothesis) against the original written nursing notes (reference).

BERTScore computes token-level semantic similarity using contextual embeddings from a pretrained BERT model, with *F*_1_ providing a balanced measure of precision and recall [26]. SBERT generates fixed-size sentence embeddings, and cosine similarity between these embeddings measures overall semantic alignment at the sentence level [27].

WER, the standard metric in ASR evaluation, calculates the minimum word-level edits required to transform the hypothesis into the reference [28]. However, its application to Korean presents challenges due to inconsistent spacing conventions. CER measures accuracy at the character level and has been recommended for Korean ASR evaluation given the language’s irregular spacing rules [28-30]. In this study, CER was calculated at the syllable level (eg, “가,” “나”) rather than the phonemic jamo level (eg, “ㄱ,” “ㅏ”) [29-31]. These 4 metrics collectively assess both semantic preservation (BERTScore and SBERT) and surface-form accuracy (WER and CER) in code-switched medical speech.

### Statistical Analysis

Statistical analyses were conducted in a Python (version 3.9.21; Python Software Foundation) environment using the pandas (version 2.3.1; pandas development team), SciPy (version 1.13.1; SciPy development team), statsmodels (version 0.14.0; statsmodels development team), and matplotlib (version 3.9.4; Matplotlib development team) libraries for data processing, analysis, and visualization. To examine the statistical significance of differences associated with temperature settings and postprocessing configurations, paired Wilcoxon signed-rank tests were used as the primary inference method, with paired Student *t* tests and bootstrap (5000 resamples) 95% CIs reported as sensitivity analyses. Paired analysis was selected because the same set of 23,652 nursing progress notes was processed through different postprocessing pipelines (baseline ASR, LLM-only postprocessing, and dictionary-augmented LLM postprocessing), and identical source audio was transcribed under different temperature settings, rendering observations across conditions statistically dependent. Effect sizes were reported as Cohen *d_z_* and matched-pairs rank-biserial *r*. To control the family-wise error rate across multiple comparisons, the Holm-Bonferroni method was applied within each comparison family (temperature sweep per model and metric; postprocessing pipeline per metric); Benjamini-Hochberg false discovery rate–adjusted *P* values are additionally provided as supplementary results.

For the temperature optimization experiments, pairwise comparisons were conducted across all 5 temperature levels (0.0, 0.2, 0.4, 0.6, and 0.8), separately for the ASR model (gpt-4o-transcribe) and for each of the 6 LLM postprocessing models. For the postprocessing pipeline, 3 pairwise comparisons were performed: (1) baseline ASR versus LLM-only postprocessing; (2) baseline ASR versus dictionary-augmented LLM postprocessing; and (3) LLM-only versus dictionary-augmented LLM postprocessing.

The level of significance was set at α=.05. All *P* values reported in the text are Holm-adjusted within each comparison family; raw and Benjamini-Hochberg–adjusted values are provided in supplementary results. Statistical significance was concluded only when both the Holm-adjusted *P* value remained below .05 and the corresponding 95% CI for the paired mean difference excluded zero.

### Ethical Considerations

This study was approved by the institutional review board (IRB) of the National Cancer Center (IRB number NCC2021-0321). All patient information in the nursing progress notes was deidentified prior to data processing. As this study used retrospective, deidentified clinical documentation, informed consent was waived by the IRB.

## Results

### Temperature Optimization

Temperature parameter optimization was conducted independently for both the ASR and postprocessing stages to identify settings that maximize transcription accuracy while maintaining output consistency.

### ASR Model

For the gpt-4o-transcribe model, temperature variations showed negligible impact on transcription performance. ASR outputs at each temperature setting were compared against the original written nursing notes. BERTScore (*F*_1_) values remained consistently around 0.913 (ranging from 0.9123 to 0.9133), while CER values stayed near 0.234 (ranging from 0.2331 to 0.2353; Table 2). Although paired Wilcoxon signed-rank tests with Holm correction detected statistically significant differences from temperature 0.0 at temperatures 0.6 and 0.8 for both BERTScore (Holm-adjusted *P*=.006 and *P*<.001, respectively) and CER (*P*=.03 and *P*<.001), the corresponding effect sizes were small (all |Cohen *d_z_*| ≤0.11), reflecting the high statistical power of N≈5000 paired comparisons rather than practically meaningful performance differences (the largest mean BERTScore difference was 0.00096, SD 0.0159 and the largest mean CER differencewas 0.00223, SD 0.0444 at temperature 0.0 vs 0.8; see Multimedia Appendix 4 for complete pairwise comparisons). Given the negligible practical effect sizes and the importance of reproducibility in clinical applications, temperature 0.0 was selected for all subsequent ASR experiments.

**Table 2.** Effect of temperature settings on automatic speech recognition performance using the gpt-4o-transcribe model^a^.

Temperature	BERTScore (*F*_1_)	CER^b^	*P* value (BERTScore)	*P* value (CER)	Selected
0	0.9133	0.2331	—^c^	—^c^	✓
0.2	0.9129	0.2339	.86	>.99	
0.4	0.913	0.2341	.58	.35	
0.6	0.9127	0.2343	.006	.03	
0.8	0.9123	0.2353	<.001	<.001	

a Only comparisons against temperature 0.0 are shown. *P* values are from paired Wilcoxon signed-rank tests with Holm correction applied across all 10 pairwise comparisons within each model and metric. Complete pairwise comparisons available in Multimedia Appendix 4.

b CER: character error rate.

c Not applicable.

### LLM Postprocessing Models

Temperature sensitivity differed markedly across the postprocessing models, and only GPT-4o showed meaningful temperature dependence. Outputs at each temperature setting were compared against the original written nursing notes. GPT-4o performed best at temperature 0.6 (BERTScore 0.9421 and CER 0.1522), with statistically significant improvements over temperature 0.0 (BERTScore 0.9391 and Holm-adjusted *P*<.001 for both BERTScore and CER); this comparison also yielded the largest temperature-related effect size observed in our analysis (Cohen *d*_z_=0.139 for BERTScore, −0.092 for CER), although the magnitude remains below Cohen threshold for a small effect, indicating that the practical impact of temperature on GPT-4o performance is modest.

In contrast, the 4 Claude variants and GPT-4.1 were largely insensitive to temperature, with all Holm-adjusted *P* values exceeding .05 except a single isolated comparison for Claude 3.7 Sonnet at temperature 0.8 CER (Holm-adjusted *P*<.001); however, the corresponding effect size for that comparison (Cohen *d*_z_=0.064) indicates a practically negligible difference (mean CER change from 0.1501, SD 0.1309 at temperature 0.0 to 0.1526, SD 0.1331 at temperature 0.8). Among these models, Claude Sonnet 4 was the strongest Claude variant, with the highest BERTScore and lowest CER (0.9444, SD 0.0429 and 0.1455, SD 0.1280, respectively, at temperature 0.0).

Model selection used CER—the metric most directly tied to character-level terminology accuracy—rather than the semantic BERTScore. This distinction mattered: GPT-4.1 attained the highest BERTScore of all models, yet not the lowest CER. GPT-4o was therefore retained as the OpenAI representative, achieving the lowest CER among the OpenAI models at temperature 0.6, where its improvement was statistically significant. Claude Sonnet 4 was selected as the Claude representative, having the lowest CER of all variants. Temperature 0.0 was kept for the temperature-invariant models to maximize reproducibility, and GPT-4o used temperature 0.6; both models then advanced to the subsequent experiments (Table 3).

**Table 3.** Comparison of postprocessing performance across large language models at different temperature settings^a^.

Model	Temperature	BERTScore (*F*_1_)	CER^b^	*P* value (BERTScore)	*P* value (CER)	Selected
GPT-4o	0	0.9391	0.1606	—^c^	—^c^	
	0.2	0.9401	0.158	<.001	.02	
	0.4	0.9398	0.1584	.02	.14	
	0.6	0.9421	0.1522	<.001^d^	<.001^d^	✓
	0.8	0.9415	0.1534	<.001^d^	<.001^d^	
GPT-4.1	0	0.9451	0.1601	—^c^	—^c^	
	0.2	0.9451	0.1567	>.99	>.99	
	0.4	0.9454	0.1534	>.99	>.99	
	0.6	0.9451	0.1537	>.99	>.99	
	0.8	0.9452	0.1564	>.99	>.99	
Claude 3.5 Sonnet v2	0	0.94	0.1839	—	—	
	0.2	0.94	0.1835	>.99	>.99	
	0.4	0.9398	0.1846	>.99	>.99	
	0.6	0.94	0.1839	>.99	>.99	
	0.8	0.9396	0.1852	>.99	>.99	
Claude 3.7 Sonnet	0	0.9435	0.1501	—	—	
	0.2	0.9435	0.1501	>.99	>.99	
	0.4	0.9434	0.1505	>.99	>.99	
	0.6	0.9431	0.1512	>.99	.44	
	0.8	0.9429	0.1526	.05	<.001^d^	
Claude Sonnet 4	0	0.9444	0.1455	—	—	✓
	0.2	0.9443	0.146	>.99	>.99	
	0.4	0.9441	0.1463	>.99	>.99	
	0.6	0.9441	0.1463	>.99	>.99	
	0.8	0.944	0.1472	>.99	.950	
Claude Opus 4	0	0.9429	0.1474	—	—	
	0.2	0.9429	0.1475	>.99	>.99	
	0.4	0.943	0.1472	>.99	>.99	
	0.6	0.9429	0.1471	>.99	>.99	
	0.8	0.9427	0.1475	>.99	>.99	

a Selected temperatures highlighted in green. GPT-4o selected at 0.6 for optimal performance; Claude Sonnet 4 selected at 0.0 for best absolute performance and reproducibility.

b CER: character error rate.

c Not applicable.

d *P*<.05 versus temperature 0.0 after Holm correction (paired Wilcoxon signed-rank test).

### ASR Performance

The baseline ASR performance using gpt-4o-transcribe with temperature 0.0 demonstrated strong initial transcription capabilities for Korean-English code-switched medical speech. ASR outputs were compared against the original written nursing notes. Across the 23,652 nursing progress notes, the system achieved a BERTScore (*F*_1_) of 0.9131 and SBERT cosine similarity of 0.8612, indicating strong semantic preservation between the ASR outputs and reference transcripts.

However, surface-form accuracy metrics revealed considerable room for improvement, with a WER of 0.3709 and a CER of 0.2336. These error rates reflect the inherent challenges of transcribing code-switched medical discourse, where English medical terms frequently appear as Korean phonetic renderings rather than standard Romanized forms. The discrepancy between semantic similarity scores (>0.86) and surface-form error rates (WER 0.37 and CER 0.23) suggests that while the ASR system successfully captured the overall meaning of utterances, it struggled with the precise orthographic representation of medical terminology.

The distribution of errors was particularly concentrated in segments containing medical abbreviations, drug names, and dosage units—elements critical for clinical documentation accuracy. Analysis of error patterns revealed that code-switching points, where speakers transitioned between Korean and English within single utterances, accounted for a disproportionate share of transcription errors. This baseline performance establishes the need for postprocessing interventions to bridge the gap between semantic understanding and clinical documentation requirements.

These baseline metrics, consistent with previous findings on code-switched ASR, establish the foundation for evaluating subsequent post-processing strategies.

### Postprocessing Performance

Postprocessing was evaluated for 2 strategies—LLM-only postprocessing and dictionary-augmented LLM postprocessing—each applied to GPT-4o and Claude Sonnet 4, with baseline ASR as the reference. All outputs were compared against the original written nursing notes.

### LLM-Only Postprocessing

Application of LLM-based postprocessing to ASR output resulted in significant performance improvements across all metrics (Table 4). GPT-4o postprocessing achieved a BERTScore of 0.9402 (SBERT 0.9266, WER 0.3160, and CER 0.1576), while Claude Sonnet 4 demonstrated slightly superior performance with a BERTScore of 0.9437 (SBERT 0.9291, WER 0.3118, and CER 0.1493), yielding improvements of 3.35%, 7.88%, 15.93%, and 36.09% over baseline.

**Table 4.** Comparison of automatic speech recognition performance metrics across processing pipelines: baseline, large language model–only, and dictionary-augmented approaches^a^.

	Baseline ASR^b^	GPT-4o		Claude Sonnet 4	
		LLM^c^-based	LLM-based	LLM-based	LLM-based
		Postprocessing	Postprocessing with dictionary	Postprocessing	Postprocessing with dictionary
BERTScore^d^ (*F*_1_)	0.9131	0.9402^e^	0.9621^e^^f^	0.9437^e^	0.9638^e^^,^^f^
SBERT^g^ cosine similarity	0.8612	0.9266^e^	0.9583^e^^f^	0.9291^e^	0.9604^e^^,^^f^
WER^h^	0.3709	0.3160^e^	0.2613^e^^f^	0.3118^e^	0.2590^e^^,^^f^
CER^i^	0.2336	0.1576^e^	0.0878^e^^f^	0.1493^e^	0.0820^e^^,^^f^

a All comparisons retained significance under both Holm and Benjamini-Hochberg correction. Effect sizes (Cohen *d_z_*) for baseline versus Dict+LLM ranged from 0.78 to 0.94 across metrics; LLM-only versus Dict+LLM ranged from 0.46 to 0.83. Full effect sizes and 95% CIs in supplementary results.

b ASR: automatic speech recognition.

c LLM: large language model.

d BERTScore: bidirectional encoder representations from transformers score.

e *P*<.05 versus automatic speech recognition baseline.

f *P*<.05 versus LLM-only postprocessing after Holm correction (paired Wilcoxon signed-rank test). Large language model–only postprocessing after Holm correction (paired Wilcoxon signed-rank test).

g SBERT: Sentence-BERT.

h WER: word error rate.

i CER: character error rate.

The reduction in CER (>32%) compared to the more modest reduction in WER (~15%) reflects the nature of the terminology normalization task, which primarily involves character-level substitutions (eg, converting Korean phonetic renderings to English terms). The relatively smaller WER improvement is partly attributed to the irregular spacing conventions of Korean, which make word-level metrics less reliable for evaluating transcription quality. Both models showed statistically significant improvements across all metrics compared to ASR-only output (paired Wilcoxon signed-rank test with Holm correction, Holm-adjusted *P*<.001 for every metric; see Table 4 and Multimedia Appendix 4 for exact *P* values for each comparison).

### Dictionary-Based Term Substitution

When applied to the full dataset of 23,652 transcribed sentences, the dictionary performed 43,507 word-level substitutions through the hierarchical matching scheme (Table 5). Exact matches accounted for the majority of replacements (32,244 instances), followed by substring matches (8190 instances). The remaining substitutions were distributed across n-gram matches (1008), space-agnostic matches (729), and single-character matches (1336). Of the 23,652 sentences, 6898 (29.17%) required no dictionary-based modifications, indicating that 16,754 (70.83%) contained at least one term requiring normalization.

**Table 5.** Distribution of dictionary-based word-level substitutions by matching rule.

Application rules	Count
Exact match	32,244
Substring match	8190
n-gram match	1008
Space-agnostic match	729
Single character match	1336
Total	43,507

The distribution of matching rules reflects the complexity of Korean-English code-switching patterns in medical discourse. Exact matches predominantly addressed common medical abbreviations and frequently used drug names, while substring and n-gram matches handled variations in Korean phonetic transcription of the same English terms. Space-agnostic matching proved essential for managing Korean’s flexible spacing conventions, particularly in compound medical terms.

These results demonstrate that dictionary-based normalization serves as an effective preliminary step for standardizing medical terminology in ASR output, providing a systematic approach to handling predictable transcription patterns before LLM-based postprocessing. The high proportion of sentences requiring modification underscores the pervasive nature of code-switching challenges in Korean medical speech recognition.

### Dictionary-Augmented Postprocessing

Integration of the medical terminology dictionary prior to LLM postprocessing yielded the highest performance across all evaluation metrics. GPT-4o with dictionary-based normalization achieved a BERTScore of 0.9621 (SBERT 0.9583, WER 0.2613, and CER 0.0878), while Claude Sonnet 4 reached a BERTScore of 0.9638 (SBERT 0.9604, WER 0.2590, and CER 0.0820).

Compared to LLM-only postprocessing, the dictionary-augmented approach demonstrated additional improvements of 2.33% in BERTScore, 3.42% in SBERT, 17.31% in WER, and 44.29% in CER for GPT-4o. Claude Sonnet 4 showed similar patterns with improvements of 2.13%, 3.37%, 16.93%, and 45.08%, respectively. These enhancements were statistically significant across all metrics (paired Wilcoxon signed-rank test with Holm correction, Holm-adjusted *P*<.001 for every metric; exact *P* values for each comparison are reported in Table 4 and Multimedia Appendix 4).

The dramatic reduction in error rates—particularly CER decreasing from 0.2336 (baseline) to 0.0820 (dictionary+Claude Sonnet 4), a 64.9% total reduction—demonstrates the synergistic effect of combining rule-based dictionary normalization with LLM-based postprocessing. The final CER below 0.10 represents a substantial improvement in transcription accuracy for medical documentation. However, clinical validation is needed to determine whether this error rate is sufficiently low for safety-critical documentation, particularly for tokens such as drug names and dosages.

**Figure 2.**
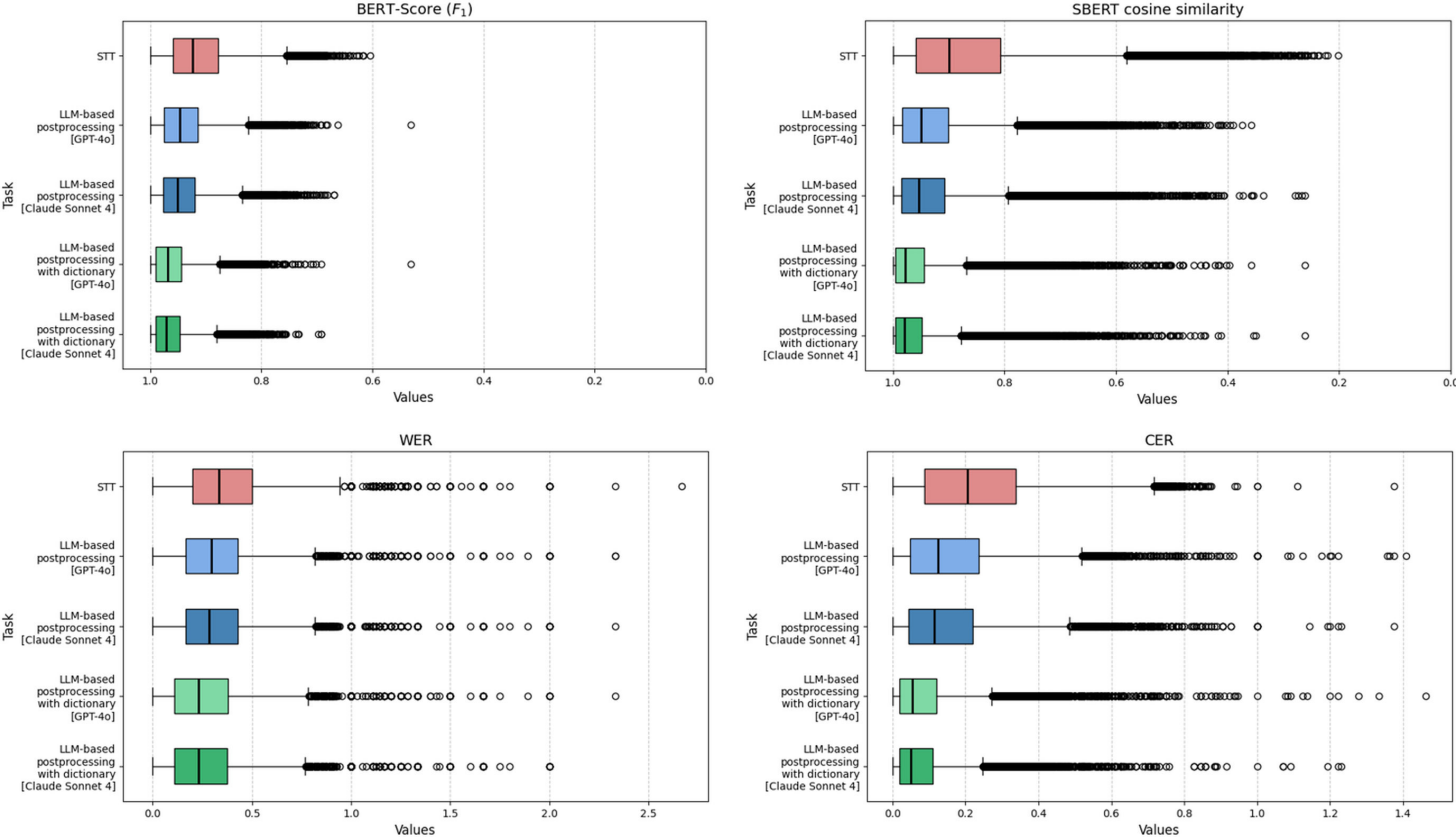
Distribution of evaluation metrics by task. CER: character error rate; LLM: large language model; SBERT: sentence-BERT; WER: word error rate.

Figure 2 illustrates the progressive improvement in performance metrics across processing stages, highlighting the complementary nature of dictionary-based and LLM-based approaches in addressing Korean-English code-switching challenges in medical ASR.

Table 4 summarizes performance across all metrics for both models and processing strategies; both strategies improved significantly over baseline ASR, and the dictionary-augmented approach achieved the highest overall accuracy.

### Subgroup Analysis of Drug-Related Utterances

Because drug names and dosages are among the most safety-critical elements of clinical documentation, we conducted a subgroup analysis restricted to utterances containing at least one drug name (n=3474). In this subset, the baseline ASR system yielded a CER of 0.3230. LLM-based postprocessing significantly reduced CER to 0.1885 with GPT-4o and 0.1619 with Claude Sonnet 4 (both Holm-adjusted *P*<.001 by paired Wilcoxon signed-rank test vs ASR baseline; exact *P* values reported in Subgroup Analysis of Drug-Related Utterances section), corresponding to relative reductions of 41.6% and 49.9%, respectively. Integrating dictionary-based normalization prior to LLM postprocessing further reduced CER to 0.0884 (GPT-4o) and 0.0837 (Claude Sonnet 4), both significant versus the ASR baseline and versus LLM-only postprocessing (Holm-adjusted paired Wilcoxon signed-rank test). The effect sizes for baseline versus dictionary-augmented CER (Cohen *d*_z_=−1.52 and −1.53) were substantially larger than the corresponding full-dataset effects, representing overall reductions of 72.6% and 74.1% from baseline. Notably, although drug-related utterances exhibited a substantially higher baseline CER than the overall dataset (0.3230 vs 0.2336), postprocessing narrowed this gap, achieving a final CER comparable to the overall result (0.0837 vs 0.0820). This subgroup result is an encouraging indication that the pipeline performs well on safety-critical content, though formal clinical validation remains necessary before deployment.

## Discussion

This study demonstrates that a hybrid approach combining medical terminology dictionary normalization with LLM postprocessing achieves significant improvements in Korean-English code-switched medical ASR. CER was reduced by 64.9% from baseline (0.2336 to 0.0820). While no universally established CER threshold for clinical acceptability currently exists in the medical ASR literature, this level of accuracy represents a substantial improvement that may support practical use in clinical documentation, pending further clinical validation. The synergistic effect of rule-based normalization and contextual refinement addresses both predictable transcription patterns and context-dependent variations. Dictionary-based normalization handles Korean phonetic renderings of English medical terms, while subsequent LLM processing resolves ambiguous cases and maintains semantic coherence. The CER reduction achieved by applying dictionary normalization before LLM processing demonstrates the value of domain-specific rule-based correction: the recurring errors here are not arbitrary but arise from a finite, standardized set of medical terms—drug names, abbreviations, and dosage units—rendered as Korean phonetic transliterations. Because this terminology is well-defined within the medical domain, a curated dictionary maps it deterministically, whereas contextual refinement alone—without guaranteed coverage of such specialized vocabulary—cannot fully resolve these patterns.

Given the bilingual nature of the dataset, we additionally evaluated performance using KR-SBERT, a Korean-optimized sentence embedding model, as a sensitivity analysis. Both embedding models indicated strong semantic alignment for the dictionary-augmented approach: KR-SBERT cosine similarity was 0.9377 (GPT-4o) and 0.9195 (Claude Sonnet 4), versus SBERT cosine similarity of 0.9583 and 0.9604, respectively. The 2 near-tied models swapped rank between metrics—Claude Sonnet 4 was marginally higher under SBERT, whereas GPT-4o was higher under KR-SBERT—but these gaps were small (≤0.02) and, because model selection in this study was based on CER rather than these semantic metrics, did not affect any conclusion. The overall finding—that dictionary-augmented post-processing yields high semantic similarity regardless of embedding model—held under both, supporting our decision to retain standard multilingual SBERT as the primary semantic-alignment metric.

Temperature optimization revealed distinct model behaviors with important clinical implications. GPT-4o achieved optimal performance at temperature 0.6 (Holm-adjusted *P*<.001 vs temperature 0.0 for both BERTScore and CER; Cohen *d*_~z_~=0.139 for BERTScore, −0.092 for CER), whereas the 4 Claude variants and GPT-4.1 showed consistent performance across all settings, with Claude Sonnet 4 performing best at temperature 0.0. Because GPT-4.1—an OpenAI model such as GPT-4o—exhibited the same temperature invariance as the Claude models, this sensitivity appears, within our limited set of models, to be specific to individual models rather than a property of a given vendor’s architecture. These behavioral differences are difficult to attribute to specific design choices, as the evaluated models span 2 vendors and differ in training cutoff, context window, and model family. GPT-4o and GPT-4.1 are OpenAI’s flagship dense multimodal models, with GPT-4.1 offering extended context and improved instruction-following over GPT-4o, whereas the 4 Claude variants share Anthropic’s alignment training paradigm (Constitutional AI), with Sonnet positioned as a balanced cost and quality tier and Opus as the highest-capability tier within each generation. Because further architectural details are proprietary and not publicly disclosed by the providers, the model-specific temperature sensitivity we observed cannot be traced to a single identifiable factor and should be regarded as an empirical characteristic of the deployed endpoints rather than a generalizable architectural property. The temperature invariance of most evaluated models makes them well-suited for deployment in health care settings where reproducibility is essential, whereas GPT-4o requires careful parameter tuning that may introduce variability in clinical workflows.

Comparison with recent literature underscores the novelty of our methodology. Kernberg et al [32] found that ChatGPT-4–generated medical notes exhibited considerable quality variability unsuitable for clinical use, whereas our structured pipeline achieves consistent, reproducible results. Similarly, while Mustafa et al [16] identified code-switching as a persistent challenge for multilingual ASR systems, our combined strategy specifically targets and mitigates these transitions through complementary mechanisms. Quantitatively, the final CER of 0.082 achieved by our pipeline is comparable to the best previously reported character-level accuracy on Korean-English medical code-switched speech. Wang et al [20] obtained a CER of 7.7% on a Korean-English medical-record corpus, but only after training an end-to-end model on 2530 hours of in-domain code-switched speech, while Lee et al [21] improved the error reduction rate by up to 17.3% through phonetic-variation modeling and language-model adaptation—both approaches requiring task-specific model training. By contrast, our pipeline reaches a similar accuracy level using a frozen general-purpose ASR model with rule-based and LLM postprocessing and no retraining. The comparison is most direct for the base ASR itself: Paik et al [19] reported that gpt-4o-transcribe, the same model used here, yields an overall mixed error rate of 21.8 on natural code-switched utterances—comparable in magnitude to our untreated baseline (CER 0.2336)—which our postprocessing subsequently reduced by 64.9%. These comparisons should be interpreted with caution, as the studies differ in error metric (CER, MER, and ERR are not directly equivalent), evaluation corpora, and recording conditions; nonetheless, they situate our results within the range of state-of-the-art Korean-English code-switched ASR while highlighting that our gains are obtained without model training.

From a clinical implementation perspective, this approach offers 3 practical advantages. First, the dictionary-based component requires no model retraining, enabling immediate deployment with existing ASR infrastructure. Second, the modular architecture allows incremental improvements: the dictionary can be expanded with institution-specific terminology while LLM components can be updated independently. Third, the dictionary-first approach constrains LLM processing to refinement rather than generation, structurally preventing the class of errors in which an English medical term is rewritten as an unrelated token, thereby reducing the risk of clinically consequential mistranscriptions. Importantly, the proposed system is intended as a documentation-assistance tool operating under a human-in-the-loop model, in which clinician review and verification remain mandatory before any transcribed note is finalized; it is designed to reduce documentation effort rather than to replace clinical oversight. These characteristics address the practical constraints of clinical environments where documentation burden contributes to physician burnout.

From a deployment perspective, several practical considerations must be addressed for clinical implementation. The current batch-processing pipeline required approximately 20 minutes per 500 audio files for ASR, which is suitable for retrospective documentation but would need optimization for real-time transcription through streaming ASR and asynchronous LLM processing. Regarding cost, application programming interface (API)-based processing incurs per-token charges; based on publicly available pricing at the time of the study, the estimated processing cost for the full dataset was approximately US $50‐75 across ASR and LLM postprocessing stages. While manageable for research, large-scale institutional deployment would require a cost-benefit analysis relative to manual documentation costs. Additionally, reliance on external commercial APIs introduces concerns about service availability, model version changes, and vendor lock-in; here too, the pipeline’s modular design mitigates these risks for institutions requiring greater operational independence.

Regarding data privacy and regulatory compliance, all data in this study were fully deidentified prior to API processing under IRB approval (NCC2021-0321), and no personally identifiable patient information was transmitted to external services. In addition, the audio recordings analyzed in this study were produced from preexisting deidentified nursing progress notes read aloud by nurses, not from live patient encounters, further reducing the risk of identifiable information being transmitted to external services. However, real-time clinical deployment would present additional privacy challenges, as reliable real-time deidentification of spoken clinical content is technically difficult. Under Korea’s Personal Information Protection Act and the Medical Service Act, transmitting patient health information to overseas API servers would require explicit consent and regulatory approval. To address these regulatory constraints, future clinical implementation should prioritize on-premise or domestic cloud-based deployment, where patient data remains within legally compliant institutional infrastructure. As noted above, the pipeline’s modular design readily supports such a transition.

This study has several limitations. First, regarding dataset constraints, the speech recordings were obtained from only 4 nurses in an acoustically isolated environment, which may not fully represent speaker diversity and acoustic variability in real clinical settings; in addition, although 5 microphone types were used in a rotating schedule to simulate device variability, ASR performance was evaluated using pooled data across all devices, and future work should include device-specific performance analysis to identify potential accuracy differences across microphone types. Relatedly, the statistical analysis treated all sentences as independent observations rather than nested within speakers (n=4) or microphones (n=5); although the paired analytical structure addresses dependence across pipelines and temperature settings for a given sentence, it does not account for residual clustering by speaker or device, which may overstate the precision of the reported *P* values and CIs. Future work should adopt mixed-effects modeling with random intercepts for speaker and device. This study also focused exclusively on nursing progress notes, requiring validation across other clinical document types.

Second, the dictionary was constructed from 1000 nursing progress notes through manual selection of mapping pairs, which may limit reproducibility and fail to capture terminology variations across different medical specialties and institutions. These 1000 notes were a subset of the 23,652-note evaluation set rather than a held-out partition; however, because the dictionary captures generalizable term-level mappings rather than note-specific content and the subset constitutes only 4.2% (1000/23,652) of the evaluation data, the resulting optimistic bias is expected to be minimal.

Third, despite achieving a CER below 0.10, the final WER remains above 25%. While much of this gap is attributable to Korean’s irregular spacing conventions rather than substantive content errors, the discrepancy means that character-level accuracy alone should be interpreted as a promising rather than a definitive indicator of clinical readiness, and word-level reliability requires further validation.

Fourth, the speech in this study was produced by nurses reading prewritten progress notes aloud, rather than spontaneously dictated during clinical care; read speech lacks the disfluencies, self-corrections, hesitations, and background noise typical of real-world dictation, and the reference transcript corresponds exactly to the read text, so the reported accuracy likely represents an optimistic upper bound, with performance on spontaneous clinical dictation remaining to be established.

Fifth, parameter optimization was limited to temperature. We chose temperature as the sole tuning parameter for 2 reasons: OpenAI’s API documentation recommends adjusting either temperature or top_p but not both, as these parameters control output randomness through overlapping mechanisms [24]; and our postprocessing task is a constrained word-level substitution rather than open-ended generation, making temperature—which directly governs the determinism of token selection—the most relevant parameter for balancing accuracy and reproducibility. Although the Anthropic API documentation exposes temperature, top_p, and top_k without an equivalent single-parameter recommendation [25], we applied the same single-parameter principle for cross-vendor consistency and a fair comparison, holding top_p (and top_k, where applicable) at provider-default values. This rationale is reinforced at the ASR stage, where temperature is in fact the only user-configurable sampling parameter exposed by the gpt-4o-transcribe end point (other generation parameters such as top_p and frequency_penalty are not exposed) [33]. At the postprocessing stage, however, joint optimization of multiple sampling parameters (eg, temperature and top_p) was not explored and may further refine performance in future work.

Finally, evaluation metrics for code-switched medical text remain suboptimal; future work should develop metrics specifically designed for multilingual medical documentation, such as spacing-normalized WER or morpheme-based error rates.

Future research directions include establishing clinically meaningful accuracy thresholds for code-switched medical ASR, as acceptable error rates may vary by clinical context and by the type of information involved (eg, drug names and dosages vs general descriptive text); conducting formal clinical validation studies to assess the real-world impact of residual errors on patient safety; expanding the dictionary to encompass additional clinical specialties and institutional variations; implementing real-time streaming ASR capabilities for immediate clinical deployment; extending this framework to other language pairs with similar code-switching patterns (eg, Japanese-English and Mandarin-English); investigating integration with existing clinical workflows and EHR systems; and exploring lightweight LLM alternatives for resource-limited settings.

This research establishes a practical framework for addressing multilingual challenges in medical ASR systems, ultimately contributing to the reduction of clinical documentation burden that underlies physician burnout. The framework’s extensibility to other language pairs and medical contexts, coupled with its compatibility with existing ASR infrastructure, positions it as a viable solution that maintains the accuracy standards essential for patient care.

## Supplementary material

10.2196/91696 Multimedia Appendix 1 System prompt configuration for the gpt-4o-transcribe automatic speech recognition model with Korean nurse persona setting.

10.2196/91696 Multimedia Appendix 2 Postprocessing prompt for GPT-4o model to normalize Korean phonetic renderings of English medical terminology.

10.2196/91696 Multimedia Appendix 3 Postprocessing prompt for Claude model variants to normalize Korean phonetic renderings of English medical terminology.

10.2196/91696 Multimedia Appendix 4 Complete pairwise statistical comparisons of temperature settings for the gpt-4o-transcribe automatic speech recognition model.

## Abbreviations

API: application programming interface
ASR: automatic speech recognition
BERTScore: bidirectional encoder representations from transformers score
CER: character error rate
EHR: electronic health record
IRB: institutional review board
LLM: large language model
SBERT: Sentence-BERT
WER: word error rate

## References

[1] (2019). Massachusetts Health & Hospital Association, Massachusetts Medical Society, a crisis in health care: a call to action on physician burnout. Patient Care Link.

[2] Berg S (2022). Burnout benchmark: 28% unhappy with current health care job. American Medical Association.

[3] Yoo JJ, Choi HB, Kim YS, Kim SG (2025). Korea’s 2024 reduction in medical research output amid physician residents’ resignation. Ewha Med J.

[4] Budd J (2023). Burnout related to electronic health record use in primary care. J Prim Care Community Health.

[5] Ng JJW, Wang E, Zhou X (2025). Evaluating the performance of artificial intelligence-based speech recognition for clinical documentation: a systematic review. BMC Med Inform Decis Mak.

[6] Bongurala AR, Save D, Virmani A, Kashyap R (2024). Transforming health care with artificial intelligence: redefining medical documentation. Mayo Clin Proc Digit Health.

[7] Vaswani A, Shazeer N, Parmar N (2017). Attention is all you need. arXiv.

[8] Zhang EY, Cheok AD, Pan Z, Cai J, Yan Y (2023). From turing to transformers: a comprehensive review and tutorial on the evolution and applications of generative transformer models. Sci.

[9] (2023). Whisper-large-v3: model card and documentation. Hugging Face.

[10] Barnes C (2023). Bringing the power of large models to Google Cloud’s speech API. Google Cloud.

[11] Tran BD, Mangu R, Tai-Seale M, Lafata JE, Zheng K (2022). Automatic speech recognition performance for digital scribes: a performance comparison between general-purpose and specialized models tuned for patient-clinician conversations. AMIA Annu Symp Proc.

[12] Kodish-Wachs J, Agassi E, Kenny P, Overhage JM (2018). A systematic comparison of contemporary automatic speech recognition engines for conversational clinical speech. AMIA Annu Symp Proc.

[13] Miner AS, Haque A, Fries JA (2020). Assessing the accuracy of automatic speech recognition for psychotherapy. NPJ Digit Med.

[14] Luo X, Zhou L, Adelgais K, Zhang Z (2025). Assessing the effectiveness of automatic speech recognition technology in emergency medicine settings: a comparative study of four AI-powered engines. J Healthc Inform Res.

[15] Roustan D, Bastardot F (2025). The clinicians’ guide to large language models: a general perspective with a focus on hallucinations. Interact J Med Res.

[16] Mustafa MB, Yusoof MA, Khalaf HK (2022). Code-switching in automatic speech recognition: the issues and future directions. Appl Sci.

[17] Ugan EY, Pham NQ, Waibel A, Waibel A DECM: evaluating bilingual ASR performance on a code-switching/mixing benchmark.

[18] Ahlawat H, Aggarwal N, Gupta D (2025). Automatic speech recognition: a survey of deep learning techniques and approaches. Int J Cogn Comput Eng.

[19] Paik G, Kim Y, Lee S, Ahn S, Kim CW (2026). HiKE: hierarchical evaluation framework for Korean-English code-switching speech recognition.

[20] Wang J, Kim J, Kim S, Lee Y Exploring lexicon-free modeling units for end-to-end Korean and Korean-English code-switching speech recognition.

[21] Lee D, Kim D, Yun S, Kim S (2021). Phonetic variation modeling and a language model adaptation for Korean English code-switching speech recognition. Appl Sci.

[22] Le-Duc K, Phan P, Pham TH MultiMed: multilingual medical speech recognition via attention encoder decoder.

[23] Adedeji A, Joshi S, Doohan B (2024). The sound of healthcare: improving medical transcription ASR accuracy with large language models. arXiv.

[24] (2025). API overview. OpenAI.

[25] (2025). Claude API docs. Messages.

[26] Hu T, Zhou XH (2024). Unveiling LLM evaluation focused on metrics: challenges and solutions. arXiv.

[27] Ganesan B, Ravikumar A, Piplani L (2024). Automated answer validation using text similarity. https://aclanthology.org/2023.icon-1.83/.

[28] (2020). Open speech analytic technologies (opensat) evaluation plan. National Institute of Standards and Technology.

[29] Bang JU, Yun S, Kim SH (2020). KsponSpeech: Korean spontaneous speech corpus for automatic speech recognition. Appl Sci.

[30] Lim Y, Kim D, Kim SH (2025). Acoustic and linguistic effects in synthesized speech augmentation for speech recognition. ETRI J.

[31] Thennal DK, James J, Gopinath DP, Ashraf K (2025). Advocating character error rate for multilingual ASR evaluation. https://aclanthology.org/2025.findings-naacl.277/.

[32] Kernberg A, Gold JA, Mohan V (2024). Using ChatGPT-4 to create structured medical notes from audio recordings of physician-patient encounters: comparative study. J Med Internet Res.

[33] (2025). Speech to text. OpenAI.

[34] (2025). ChatGPT. OpenAI.

