# Supplementary material for: Dictionary-Augmented Large Language Model Postprocessing for Bilingual Code-Switched Medical Speech Recognition: Development and Evaluation Study
Source: J Med Internet Res. 2026 Jul 8;28:e91696. doi: 10.2196/91696 (PMC13344086; doi:10.2196/91696)
Supplement: Multimedia Appendix 1 [file jmir-v28-e91696-s001.docx]

**Multimedia Appendix 1**. System prompt configuration for the gpt-4o-transcribe automatic speech recognition model with Korean nurse persona setting

“This audio was recorded by a Korean nurse in a university hospital ward in South Korea to document nursing records.

The content includes both Korean and English terms.

Transcribe exactly as spoken, without inferring or translating any words.

Do not add, omit, or interpret meanings.

Please pay close attention to Korean-accented English and Korean pronunciation patterns.

Apply consistent spacing for Korean words, avoiding inconsistencies in spacing across the transcription.”
