# Supplementary material for: Dictionary-Augmented Large Language Model Postprocessing for Bilingual Code-Switched Medical Speech Recognition: Development and Evaluation Study
Source: J Med Internet Res. 2026 Jul 8;28:e91696. doi: 10.2196/91696 (PMC13344086; doi:10.2196/91696)
Supplement: Multimedia Appendix 4 [file jmir-v28-e91696-s004.docx]

**Multimedia Appendix 4.** Complete pairwise statistical comparisons of temperature settings for the gpt-4o-transcribe automatic speech recognition model

| **Temperature (Temp)** | **BERTScore (*F*_1_)** | **CER** | ***P*-value (BERTScore) vs:** | | | | | ***P*-value (CER) vs:** | | | | | |
| --- | --- | --- | --- | --- | --- | --- | --- | --- | --- | --- | --- | --- | --- |
|  |  |  | **Temp 0.0** | **Temp 0.2** | **Temp 0.4** | **Temp 0.6** | **Temp 0.8** | | **Temp 0.0** | **Temp 0.2** | **Temp 0.4** | **Temp 0.6** | **Temp 0.8** |
| 0 | 0.9133 | 0.2331 | - | - | - | - | - | | - | - | - | - | - |
| 0.2 | 0.9129 | 0.2339 | 0.722 | - | - | - | - | | 0.812 | - | - | - | - |
| 0.4 | 0.913 | 0.2341 | 0.795 | 0.922 | - | - | - | | 0.767 | 0.954 | - | - | - |
| 0.6 | 0.9127 | 0.2343 | 0.617 | 0.886 | 0.809 | - | - | | 0.710 | 0.894 | 0.940 | - | - |
| 0.8 | 0.9123 | 0.2353 | 0.417 | 0.649 | 0.580 | 0.755 | - | | 0.504 | 0.667 | 0.709 | 0.767 | - |
